# RSK4 promotes the metastasis of clear cell renal cell carcinoma by activating RUNX1-mediated angiogenesis

**DOI:** 10.1080/15384047.2025.2452025

**Published:** 2025-01-10

**Authors:** Jing Ma, Yanru Yang, Kaijing Wang, Jin Liu, Junyi Feng, Gongcheng Wang, Shuangping Guo, Linni Fan

**Affiliations:** a State Key Laboratory of Cancer Biology, Department of Pathology, Xijing Hospital, Air Force Military Medical University, Xi’an, China; b Basic Medical Research Experimental Center, Yan’an University of Medicine, Yan’an, China

**Keywords:** RSK4, metastasis, angiogenesis, clear cell renal cell carcinoma

## Abstract

Ribosomal S6 protein kinase 4 (RSK4), a member of the serine‒threonine kinase family, plays a vital role in the Ras‒MAPK pathway. This kinase is responsible for managing several cellular activities, including cell growth, proliferation, survival, and mobility. In this study, we observed higher RSK4 protein expression in clear cell renal cell carcinoma (ccRCC) than in normal kidney tissue, and the overexpression of RSK4 might predict poor outcomes for ccRCC patients. Notably, renal cell carcinoma (RCC) is rich in blood vessels; therefore, this study aimed to explore the biological function of RSK4 in ccRCC progression and its specific regulatory mechanism. We analyzed changes in the expression of target genes through transcriptomic and proteomic assessments. We also conducted tube formation assays and VEGF ELISAs to understand the role of RSK4 in angiogenesis. Additionally, we evaluated the regulatory effect of RUNX1 on EPHA2 transcription using a luciferase reporter gene assay and observed that the effect of RUNX1 on activating EPHA2 transcription was negated after the binding site was mutated. Our findings suggested that RSK4 enhanced tube formation by stimulating VEGF secretion. Concurrently, in vivo experiments confirmed that RSK4 expedited RCC metastasis and angiogenesis. This evidence indicates that RSK4 may serve as a new prognostic marker and play a vital role in RCC metastasis.

## Introduction

Renal cell carcinoma (RCC), noted for its significant vascular nature, is one of the most common types of urological malignancies, and its persistent prevalence continues to present a formidable challenge to urological research and treatment efforts.^1^ This issue becomes increasingly complicated when considering the diverse histological subtypes of RCC, among which ccRCC has emerged as the most frequently observed subtype.^2^ As ccRCC progresses to advanced stages, the tumor frequently metastasizes, an ominous transition that unfortunately correlates with substantially poor clinical prognoses.^3,4^ In terms of clinical research, the disease course of RCC is still unclear in the majority of cases.^5^ At present, various molecular pathways related to the malignant progression of RCC have been identified, including the VHL/HIF pathway,^6^ the MAPK pathway, the Wnt/β-catenin pathway,^7^ etc. The inactivation of VHL increases the activity of hypoxia-induced factor (HIF), which is essential for angiogenesis, anaerobic processes, metabolism, inflammation, and metastasis.^6^ Furthermore, RCC presents a unique therapeutic challenge, as it is notoriously resistant to traditional treatment options such as radiation and chemotherapy.^8^ Thus, the discovery of novel biological markers that improve early diagnosis and patient therapy is essential.

Ribosomal S6 protein kinases (RSKs), which belong to the class of serine-threonine kinases, are critical components of the Ras – mitogen-activated protein kinase (MAPK) signaling cascade. Their functions span a wide range of cellular activities, such as promoting cell growth, facilitating cell proliferation, ensuring cell survival, and regulating cellular motility.^9^ Ribosomal S6 protein kinase 4 (RSK4), a member of the RSK family, has drawn significant attention in this context. Many studies have investigated the patterns of RSK4 mRNA expression across human tissues, providing key insights into its role under various physiological and pathological conditions.^10^ From this body of work, RSK4 has emerged as a potential candidate tumor suppressor gene, as evidenced by several scientific reports. Certain investigations have highlighted a decrease in RSK4 expression in a subset of cancers, whereas others have reported that upregulated RSK4 expression appears to promote the invasive and metastatic tendencies of tumor cells. Curiously, the scenario in RCC appears to deviate from the norm. RSK4 expression in RCC tissue appears to be significantly higher than that in normal kidney tissue. Moreover, a higher incidence of invasive and metastatic behaviors has been noted in RCC tumors overexpressing RSK4. These observations suggest that RSK4 may play an instrumental role in driving the progression of RCC tumors.^11^ Despite these intriguing findings, the exact functions and influences of RSK4 in the context of RCC remain a topic of active research and are yet to be fully elucidated.

Among the many transcription factors, Runt-related transcription factor 1 (RUNX1), also referred to as acute myeloid leukemia 1, is notable. It is part of the broader RUNX family of transcription factors, a lineage that also includes genes such as RUNX2 and RUNX3. The RUNX family is marked by evolutionary conservation and is required for the tissue-specific lineage commitment across a range of biological entities.^12^ RUNX1 has received attention for its potential role in modulating angiogenesis, a critical process in tumor growth and metastasis.^13^ However, the relationship between RSK4, a kinase with a significant influence on cell behavior, and RUNX1, especially in the context of RCC, is not yet fully understood.

EPHA2 plays a substantial role in the broad landscape of the EPH receptor tyrosine kinase family, particularly in relation to the metastasis of a wide spectrum of cancers. Emerging research illustrates that EPHA2 is a critical activator of AMPK signaling through the Ephrin A1-facilitated forward pathway.^14^ This activation can potentially amplify the tubulogenic and migratory activities of endothelial cells.^15^ Initially identified for its role in the migration of neurons during embryonic development, EphA2 has been extensively studied in the context of angiogenesis.^16^ It is known to participate in various vital processes, including the migration of endothelial cells, the assembly of vascular structures, and the regulation of junctions between epithelial cells.^17,18^ Moreover, EphA2 is highly expressed in ccRCC and promotes the migration and invasion of ccRCC cells.^19^

The focus of our study was to explore the blood vessel density within RCC and decipher the potential underlying mechanisms, relying on RCC cell lines as the primary research tools. Intriguingly, our data highlight a probable relationship between increased RSK4 levels and the aggressive phenotype often observed in RCC. This study revealed that the overexpression of RSK4 can stimulate an increase in the secretion of vascular endothelial growth factor (VEGF) and increase tube formation efficiency. Moreover, the overexpression of RSK4 also facilitates the phosphorylation of RUNX1, which subsequently leads to the upregulation of EPHA2, a gene that plays a pivotal role in angiogenesis downstream. Collectively, our results contribute to a broader comprehension of the mechanisms driving RCC tumorigenesis, highlighting potential molecular pathways involving RSK4, RUNX1, and EPHA2 in the specific context of RCC cell lines. This improved understanding could pave the way for further in-depth investigations and the development of possible therapeutic interventions in the future.

## Results

### RSK4 is highly expressed in ccRCC and is associated with poor survival of ccRCC patients

**Figure 1.**
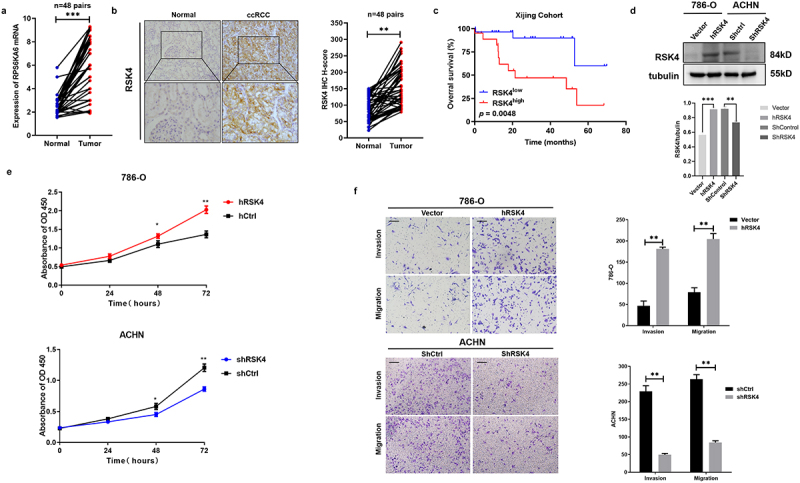
RSK4 is upregulated in ccRCC and predicts a poor prognosis.

An analysis of 48 paired ccRCC and adjacent nontumor tissues revealed significantly higher RSK4 mRNA and protein levels in ccRCC tissues than in their corresponding nontumor tissues (Figure 1a–b). Importantly, compared with low RSK4 expression, high RSK4 expression was correlated with shorter overall survival (OS) of patients with ccRCC.While this study opens new avenues for research, it also highlights the importance of a comprehensive understanding of the role of RSK4 in RCC (Figure 1c and Table 1).

(A) RPS6KA6 mRNA levels in 48 pairs of RCC samples and adjacent nontumor tissues were determined by real-time PCR. (B) Representative images of IHC staining and H-scores for RSK4 protein expression in RCC tumor tissues and adjacent nontumor tissues. Scale bars: 100 μm. (C) Kaplan‒Meier estimates of the OS of RCC patients in the Xijing cohort based on the RSK4 expression levels. (D) Overexpression and knockdown of RSK4 were confirmed at the protein level in 786-O and ACHN cells. (E) CCK-8 tests of the proliferation level in 786-O and ACHN cell lines. (F) Overexpression of RSK4 significantly promoted the migration and invasion of 786-O cells. (F) Knockdown of RSK4 significantly inhibited the migration and invasion of ACHN cells. Bar, 100 μm. The data are presented as the means ± SDs of three independent experiments, **p < .01 and ***p < .001.

**Table 1.** Univariate and multivariate analysis of overall survival in RCC patients.

				Univariate analysis		Multivariate analysis		
Variables			Categories	HR (95% CI)	p-value		HR (95% CI)	*p*-value
Age			≥60/< 60	1.24 (0.36–1.436)	.765		1.346 (1.245–2.723)	.2444
WHO/ISUP grade			1–2/3–4	3.24 (0.87–12.09)	***0.014***		1.68 (0.47–5.98)	***0.044***
Gender			Male/Female	1.7 (0.46–4.52)	.3979		1.907 (1.364–2.251)	.9734
RSK4 expression			High/Low	2.858 (1.352–5.849)	.0036		3.793 (1.358–5.859)	.0069

Given the body of evidence supporting the significant correlation between RSK4 overexpression and cancer metastasis, coupled with the importance of cell migration and invasion in tumor metastasis, further investigations of the biological role of RSK4 in ccRCC are crucial. Therefore, we investigated the relationship between RSK4 expression and tumor metastasis. We first assessed the effect of RSK4 on the migration and invasion of RCC cells in vitro and then established an RSK4-overexpressing 786-O cell line and a RSK4-knockdown ACHN cell line (Figure 1d). The RSK4`s capability of affecting the proliferation of RCC cells received evaluation. RSK4-overexpressing 786-O cells confirmed significant enhancement in comparison to proliferation the vector control cells. However, cells in infection with shRSK4 told a dramatic decrease on cell proliferation (Figure 1e). We proceeded to gauge the effects of RSK4 on the migratory and invasive activities of RCC cells by performing transwell assays. The results revealed that forced expression of RSK4 increased the migration and invasion of 786O cells compared with the control group. In contrast, shRSK4-treated ACHN cells displayed marked reductions in both invasion and migration activities (Figure 1f). While these results suggest new avenues for research, they also highlight the importance of a comprehensive understanding of the role of RSK4 in RCC.

## RSK4 overexpression enhanced angiogenesis in vitro

**Figure 2.**
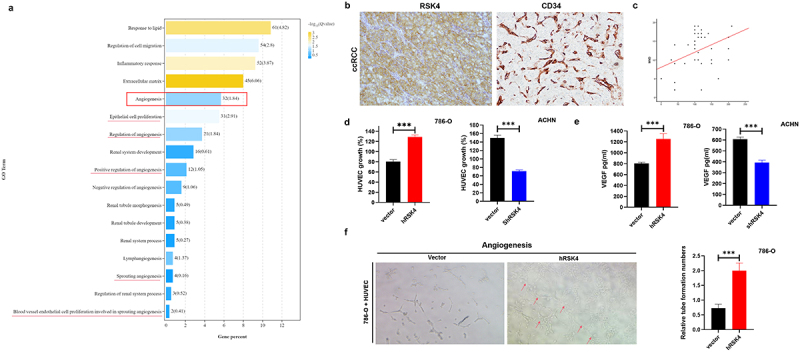
RSK4 expression is closely correlated with the ccRCC microvessel density.

Renal cell carcinoma (RCC), the most common malignant urological tumor, is recognized by its abundant vasculature. Given that the correlation between high RSK4 expression and cancer metastasis is significant and that migration and invasion abilities are crucial for tumor metastasis, we investigated the biological function of RSK4 in RCC. We conducted an untargeted multiomics study involving both transcriptomics and proteomics in wild-type (WT) and RSK4-overexpressing 786-O cells to elucidate the intricate mechanism of RSK4. The Gene Ontology (GO) analysis indicated that the DEGs were enriched mainly in angiogenesis and vascular development (Figure 2a), indicating an angiogenic role for RSK4 in RCC. IHC staining of the RSK4 and CD34 proteins revealed that the microvessel density (MVD) was increased in RCC (Figure 2b); the correlation analysis revealed a positive correlation between RSK4 expression (H score^20^) and the MVD (Figure 2c, *p* = .009), suggesting that RSK4 may be involved in angiogenesis in ccRCC.

(A) Gene Ontology (GO) analysis of DEGs. (B) The MVD was higher in ccRCC tissues. (C) The expression of RSK4 in RCC was positively correlated with the MVD (*P* = 0.009). (D) RSK4 overexpression significantly increased HUVEC growth compared with that of the control group, and vice versa. (E) ELISA was performed to determine the levels of VEGF secreted by hRSK4-expressing 786-O and shRSK4-transfected ACHN cells. (F) Representative images of in situ tube formation after treatment with the supernatant of hRSK4-expressing 786-O cells. The arrow indicates the formed tubule. The data are presented as the means ± SDs of three independent experiments; ***P < 0.001.

Vascular endothelial growth factor (VEGF), a key modulator predominantly expressed in endothelial cells, is instrumental in the intricate process of angiogenesis. Consequently, we investigated whether RSK4 affects angiogenesis in vitro. The angiogenic potential of the supernatants of 786-O and ACHN cells transfected with RSK4 was determined by performing endothelial cell proliferation and tube formation assays. In the cell proliferation assay, we found that the conditioned medium from the 786-O cells transfected with hRSK4 promoted the proliferation of endothelial cells in contrast to that from the vector-transfected cells, and vice versa (Figure 2d). Then, we conducted an ELISA to confirm the role of RSK4 in regulating VEGF expression (Figure 2e). In the tube formation assay, the average number of complete tubular structures formed by the HUVECs was significantly increased after treatment with the conditioned medium from RSK4-overexpressing cells compared with that from the control cells (Figure 2f). These findings suggest a potential role for RSK4 in promoting angiogenesis in RCC.

## RSK4 promotes metastasis in ccRCC and is closely correlated with angiogenesis in vivo

**Figure 3.**
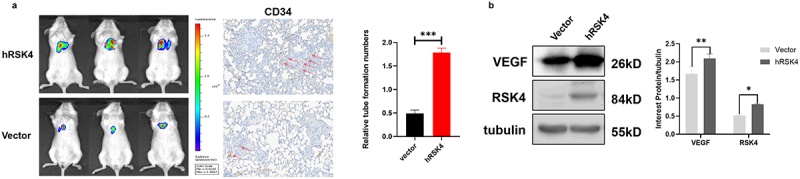
RSK4 promotes metastasis in ccRCC and is closely correlated with the microvessel density in vivo.

We overexpressed RSK4 in luciferase-expressing 786-O RCC to further substantial the putative anticancer properties of RSK4 in a live model. Next, we employed LLC-luc-vector and LLC-luc-hRSK4 to establish NOD/SCID mouse models, providing a platform to scrutinize the role of RSK4 in tumor metastasis. After 6 weeks, bioluminescence imaging was used to measure the metastatic lesions (Figure 3a). The survival rates recorded for the RSK4-overexpressing group were lower than those recorded for the control group (Figure 3b). Moreover, the Western blot analysis revealed increased VEGF expression in RCC tumors from mice injected with hRSK4-expressing cells (Figure 3c). Further analysis via IHC for CD34 in metastatic tumors revealed an increased MVD in hRSK4-expressing tumors (Figure 3d).

(A) Cells stably expressing LLC-luc-vector and LLC-luc-hRSK4 were injected into NOD/SCID mice through the tail vein. Bioluminescence images are shown. IHC analyses of CD34 levels in the indicated groups; the arrows indicate microvessels. (B) Western blot of RSK4 and VEGF levels in RCC tumors from mice injected with vector-transfected or hRSK4-transfected cells. The data are presented as the means ± SDs; ***P < 0.001.

## RSK4 enhances RCC angiogenesis by regulating RUNX1 and EPHA2

**Figure 4.**
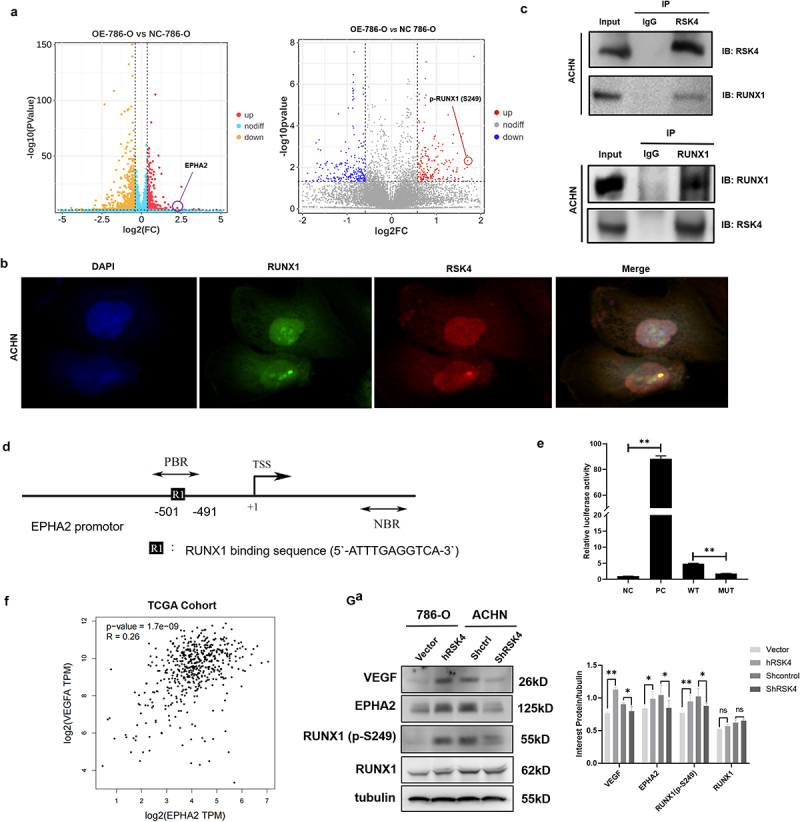
RSK4 interacts with RUNX1 in RCC, and EPHA2 is a RUNX1 target gene.

An untargeted multiomics analysis using transcriptomics and proteomics of wild-type (WT) and RSK4-overexpressing 786-O cells was performed to obtain a comprehensive view of the complex mechanisms of RSK4. This approach facilitated the identification of 617 differentially expressed genes (DEGs), defined by an absolute fold change > 1.5 and a false discovery rate (FDR) less than 0.05. Among them, EPHA2 was significantly upregulated (Figure 4a, left panel). As RSK4 is a serine/threonine protein kinase, our next step was to detect potential targets phosphorylated by RSK4. The proteomic analysis revealed that Ser-249 of RUNX1 was the most notable phosphorylation site, prompting us to concentrate on this site in subsequent investigations (Figure 4a right). In addition, the colocalization of activated RUNX1 and RSK4 was examined by immunofluorescence staining and microscopy in ACHN cells (Figure 4b). We employed an endogenous coimmunoprecipitation assay to further substantiate this interaction, and the results confirmed the direct binding of RSK4 to RUNX1 in the ACHN RCC cell line (Figure 4c). Transcription factor predictions revealed a potential RUNX1 binding site between positions −501 and −491, which is located upstream of the transcriptional initiation site in the EPHA2 promoter (Figure 4d). The regulatory effect of RUNX1 on EPHA2 transcription was evaluated through a luciferase reporter assay, and mutation of the binding site reversed the ability of RUNX1 to induce EPHA2 transcription (Figure 4e). Additionally, we conducted an analysis to investigate the relationship between VEGF and EPHA2 expression in ccRCC using GEPIA (Figure 4f). The western blot analysis revealed that RSK4 overexpression led to a significant increase in the levels of RUNX1 (Ser249) phosphorylation and EPHA2 compared with those in the control group, and vice versa (Figure 4g). Collectively, these findings underscore a significant correlation between RSK4 and RUNX1 in RCC, positioning EPHA2 as a target gene of RUNX1.

(A) Volcano plot illustrating the distributions of DEGs in WT and RSK4-overexpressing 786-O cells. RSK4 overexpression significantly increased the levels of EPHA2 and RUNX1 (Ser249) phosphorylation compared with those in the control group. (B) The colocalization of activated RUNX1 and RSK4 in ACHN cells was examined by immunofluorescence staining and microscopy. Scale bars = 10 μm. (C) RSK4 binds to RUNX1 in ACHN cells. Endogenous RSK4 was immunoprecipitated from ACHN cells and then probed with an anti-RUNX1 antibody. Immunoglobulin G (IgG) served as a negative control. (D) Predicted binding sites for RUNX1 in the promoter region of EPHA2. (E) The regulatory effect of RUNX1 on EPHA2 transcription was detected via a luciferase reporter assay. Abbreviations: NC, negative control; PC, positive control; WT, wild-type; MUT, mutant. (F) Correlations between VEGF and EPHA2 expression in ccRCC were analyzed using GEPIA (http://gepia.cancer-pku.cn/detail.php). (G) Western blots showing the levels of VEGF, EPHA2, RUNX1, and p-RUNX1 (S249) in ccRCC cell lines. The data are presented as the means ± SDs of three independent experiments, **P<0.01.

## RSK4 inhibition with BI-D1870 attenuates tube formation and tumor growth

**Figure 5.**
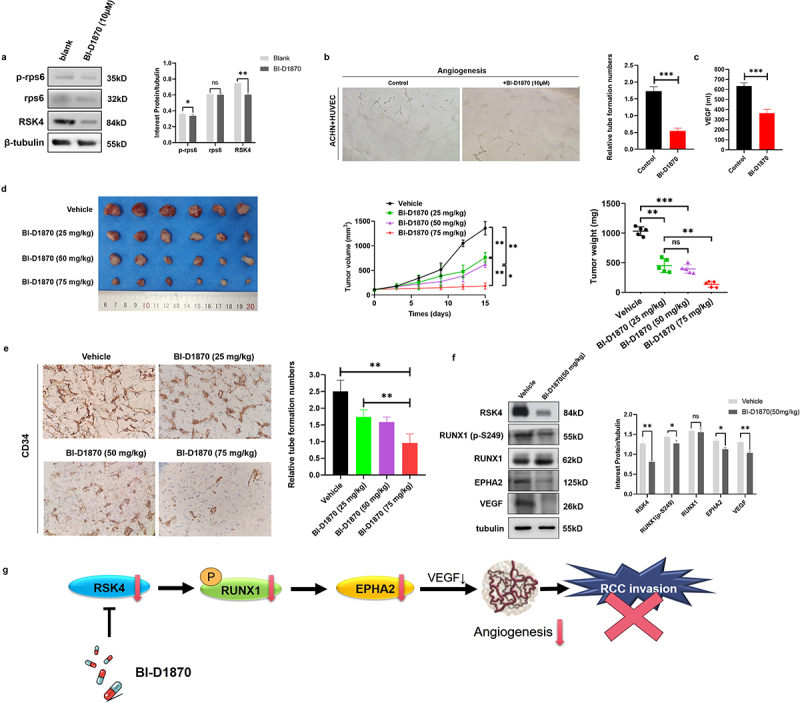
BI-D1870 inhibits tumor growth and tube formation in vivo.

We further verified the role of RSK4 in ccRCC angiogenesis by detecting the phosphorylation of MAPK using the rps6 antibody in ACHN cell lines expressing endogenous RSK4 after treatment with the RSK inhibitor BI-D1870 (Figure 5a). In the tube formation assay, the average number of complete tubular structures formed by the HUVECs was significantly decreased after exposure to the conditioned medium from the experimental group (Figure 5b). The secretion of VEGF by ACHN cells also decreased following BI-D1870 treatment (Figure 5c). We verified the above in vitro data in vivo by inoculating mice with different cells. Compared with those from the BI-D1870 treatment group, tumors from the control group were much larger, and the tumor weight statistics were similar (Figure 5d). The IHC analysis of the CD34 protein revealed that the microvessel density (MVD) decreased in xenograft tumors subjected to the indicated treatments (Figure 5e). Western blot analyses of the tumor samples from the control and BI-D1870 (50 mg/kg) groups of mice revealed higher p-RUNX1 (S249), EPHA2 and VEGF levels in the control tumors (Figure 5f). These data revealed that the tumorigenic properties and microvessel density of the experimental group were significantly reduced, suggesting that RSK4 is essential for ccRCC invasion via the activation of RUNX1-EPHA2-VEGF-mediated angiogenesis (Figure 5g).

(A) BI-D1870 significantly inhibited RSK4 expression in ACHN cells. (B-C) BI-D1870 significantly inhibited tube formation and VEGF secretion. (D) RCC-derived xenografts in mice treated with the vehicle control or 25 mg/kg/day, 50 mg/kg/day, or 75 mg/kg/day BI-D1870. The growth curves for the tumor size and average tumor weight are presented (*n* = 6 mice each). (E) Representative images of IHC staining showing CD34 protein expression and relative numbers of tubes formed in xenograft tumors from mice administered the indicated treatments. (F) Western blots showing the levels of RSK4, RUNX1, p-RUNX1 (S249), EPHA2 and VEGF in xenograft tumors treated with the vehicle control or BI-D1870 (50 mg/kg). (G) Graphical abstract showing that RSK4 promotes metastasis in clear cell renal cell carcinoma by activating RUNX1-mediated angiogenesis. **P<0.01 and *** *P* < 0.001.

## Discussion

RCC is a commonly encountered malignancy within the field of urology and is characterized by an abundant vascular network.^21^ Contemporary research has provided insights into numerous molecular pathways associated with disease progression, including the VHL/HIF pathway,^6^ the MAPK pathway, and the Wnt/β-catenin pathway.^7^ The loss of VHL function activates hypoxia-induced factor (HIF), a critical component in processes such as angiogenesis, anaerobic metabolism, inflammation, and metastasis.^22–25^ Investigating the pathogenesis and metastatic behavior of RCC has allowed scientists to explore diagnostic approaches and therapeutic interventions while also examining molecules linked with metastasis.^26^ The ultimate objective is to devise strategies that can predict the patient prognosis with a high degree of accuracy.

RSK4, a member of the ribosomal S6 kinase (RSK) family, largely remains an enigma.^27^ Previous studies have highlighted a significant increase in the RSK4 level in RCC compared with that in normal kidney tissue. This increased expression appears to be associated with a greater tendency toward invasiveness and metastasis, suggesting the potential significance of RSK4 in the progression of RCC.^9^

Our previous investigations revealed high expression of RSK4 in various cell types, including pancreatic ductal epithelial cells, salivary epithelial cells, sweat gland epithelial cells, and B lymphocytes located in tonsil germinal centers.^11^ Additionally, various malignancies, such as clear cell RCC, uterine clear cell carcinoma, ovarian serous papillary cystadenocarcinoma, and gastric adenocarcinoma, exhibit high RSK4 expression.^9^ However, certain cancer types, such as breast cancer and hepatocellular carcinoma, present only weak RSK4 positivity.^11,28,29^ Despite these findings, the exact functions and implications of RSK4 in RCC remain unclear. This lack of clarity highlights the need for an in-depth exploration of the etiology and molecular mechanics of RCC, along with the pursuit of more precise and targeted therapeutic strategies.

Our study reveals previously unknown aspects of the pivotal role of RSK4 in RCC, especially in the context of disease progression, angiogenesis and metastasis. We observed a striking increase in the migratory and invasive capacities of RCC cells in response to RSK4 overexpression. This increased activity mirrors the function of RSK4 in vivo. Additionally, a clear increase in tube formation by HUVECs was observed when RSK4 was overexpressed.

Our findings provide a novel illustration of how RSK4 overexpression profoundly amplifies the invasion and angiogenesis of RCC cells. These conclusions are drawn from a combination of in vitro and in vivo experimental techniques. The exact molecular pathway that RSK4 utilizes to modulate invasiveness and angiogenesis, however, remains elusive. Current research suggests the crucial involvement of RUNX1 and EPHA2 in angiogenesis and the invasion of cancer cells.^30^

Runt-related transcription factor 1 (RUNX1), also referred to as acute myeloid leukemia 1, belongs to the family of RUNX transcription factors and shares a group with RUNX2 and RUNX3. The crucial roles of RUNX1 extend across definitive hematopoiesis, *T*- and B-cell lineage specification, and the development of neurons, highlighting its relevance.^31,32^ Additionally, the orchestration of chondro-osteoblast differentiation, which is pivotal in osteogenesis, is managed by RUNX2.^33^ Neurogenesis in the dorsal root ganglia and the proliferation of the gastrointestinal epithelium involve the function of RUNX3.^34^ RUNX family members also play roles in modulating carcinogenesis, with irregularities and mutations linked to various types of neoplasms.^35^ While the regulatory function of RUNX1 in angiogenesis is well established,^13^ its association with RSK4 in RCC remains unknown. EPHA2 is typically found on the membrane of epithelial cells in healthy tissues and interacts with its ligand, Ephrin A1, which is located on the surface of neighboring cells.^15^ The mechanism of EPHA2 in cancer progression is complex; elevated EphA2 concentrations are associated with a poor prognosis for esophageal cancers and glioblastomas and promote the growth and spread of several malignant tumor cells.^36–39^ Additionally, EphA2 is highly expressed in ccRCC, increasing the migration and invasion capabilities of ccRCC cells.^19^

In our research, we discerned a marked increase in both RUNX1 phosphorylation and EPHA2 levels following the overexpression of RSK4. Initially identified as a vital molecule in neuronal migration during embryogenesis, the role of the EphA2 receptor in angiogenesis has been the subject of extensive scrutiny. It is associated with an array of processes, such as the movement of endothelial cells, the formation of the vasculature, and the control of epithelial cell junctions.^40^ Tumor growth mainly relies on the induction and maintenance of a blood supply,^41^ where angiogenic factors, such as VEGF, exert a significant influence. Given the pivotal role of VEGF in tumor angiogenesis, we investigated the effects of RSK4 overexpression on VEGF activity. The data collected revealed an increase in VEGF secretion following RSK4 overexpression in RCC cells. The subsequent promotion of RCC angiogenesis coincided with the increased expression of VEGF and signaling through the RSK4/RUNX1/EPHA2 pathway (Figure 5G).

In summary, our investigation has provided insights into a new signaling pathway in RCC, RSK4/RUNX1/EPHA2. The activation of this pathway amplifies RCC angiogenesis and promotes tumor metastasis both within live models and in laboratory settings. These insights underscore the potential of RSK4 as a valuable therapeutic target specifically for RCC, with a significant contribution to addressing RCC metastasis. Future research will focus on exploring the role of RSK4 in regulating VEGF expression, as well as the specific implications of RSK4 phosphorylation.

## Materials and methods

### Clinical samples

In the course of our investigation, we used primary ccRCC tissue samples, which were meticulously collected from a patient pool of 100 individuals. Initial surgical procedures facilitated the collection of these samples at the renowned Xijing Hospital, an affiliate of the Air Force Medical University, Xi’an, China. This phase of tissue collection occurred over an extensive period from 2018 to 2022. We performed in-depth investigations of various sources of information to compile a comprehensive dataset. Detailed reports of clinicopathological outcomes were derived from a careful examination of both surgical documentation and methodical evaluations of pathological records. Besides, written informed consent was obtained from all individual participants included in the study.

### Immunohistochemistry and H-scores

The methods used for IHC and the evaluation of the RSK4 h score can be found in our previous study.^20^

### Cell lines, plasmids and transfection

In the context of our research, we employed ACHN cells, a human RCC cell line, which were secured from the Experimental Animal-raising Center at the Air Force Military Medical University situated in Xi’an, China. These particular cells were then subjected to carefully controlled conditions and cultured in enriched RPMI 1640 medium (HyClone, Thermo, USA) supplemented with 20% fetal bovine serum (FBS; Gibco, Carlsbad, CA, USA). Our team ensured that the cells were cultured under the optimal conditions of 37°C with an atmosphere composed of 5% CO_2_. Our aspiration to induce the overexpression of human RSK4 guided our choice of the pcDNA3.1/Neo-RSK4 plasmid as an effective tool for enabling stable transfection of the RCC cell line. For transfection, we used Lipofectamine 2000 (Invitrogen, Carlsbad, CA, USA) and strictly following the manufacturer’s guidelines. Upon reaching an estimated 90% confluence within the six-well plates, the cells were transfected. A total of 4.0 mg of plasmid DNA was mixed with 10 ml of Lipofectamine 2000 per well. After transfection, a rest period of 48 hours was allowed before the next step in the process. The cells were subsequently trypsinized and replated onto larger 10-cm culture dishes. As a complement to this procedure, we introduced 300 mg/ml G418 (Gibco), which significantly contributed to the isolation and expansion of individual cell clones. We validated the expression of RSK4 at both the mRNA and protein levels by meticulously analyzing each clone using Western blotting and reverse transcription‒PCR.

### RNA extraction and quantitative real-time PCR (qRT‒PCR)

Using TRIzol (Invitrogen), total RNA was extracted from both freshly obtained tissues and cells after transfection. The extraction process adhered to the manufacturer’s instructions, with the extracted RNA subsequently stored at a temperature of −80°C for optimal preservation. We were able to identify the presence of the RSK4 mRNA in stable transfectants via reverse transcription‒PCR using a SYBR Green II kit (Takara, Shiga-ken, Japan). GAPDH served as the endogenous reference gene in this procedure. The specific primers used for RSK4 were 5’-TGAGTGGTGGAAACTGGGACAATA-3’ (forward) and 5’-TGGCATGGACTGTGGTCATGAGTC-3’ (reverse). For GAPDH, the primers used were 5’-GCACCGTCAAGGCTGAGAAC-3’ (forward) and 5’-TGGTGAA GACGCCAGTGGA-3’ (reverse). The annealing process was meticulously performed at a regulated temperature of 60°C to ensure the successful binding of the primers to their respective templates.

### Western blot analysis

For cell lysis, we utilized a specific buffer containing 1–5 mg/ml aprotinin, 1–5 mg/ml leupeptin, and other essential components. With the aid of a BCA protein assay (Pierce, Thermo), the protein concentrations were accurately assessed. Subsequently, protein aliquots, each containing 50 mg of protein, were separated on 10% SDS‒PAGE gels and transferred onto PVDF membranes (Millipore, Billerica, MA, USA). We employed chemiluminescence (Pierce, Thermo) for visual detection. Our analysis included various primary antibodies, specifically, rabbit anti-RSK4 (Sigma), rabbit anti-RUNX1, rabbit anti-phosphorylated S249, rabbit anti-EPHA2 (Cell Signaling Technology), and rabbit anti-VEGFA (Proteintech), facilitating a comprehensive investigation of the objectives of our study.

### Growth curve analysis

In a 6-well plate, the cells were plated at the cell size of 2.5 × 105 per well and exposed to AG490 at a concentration of 100 μM. Finally, a hemocytometer was used to count in triplicate at different time points to generate a growth curve. In a 96-well plate, the cells were plated at 100 μl per well containing 2500 or 5000 cells, with or without exposure to OTS964 (25 nM). Finally, add 10 μl of Cell Counting Kit-8 (TargetMol, Boston, MA, USA) to each well to detect the absorbance at 450 nm.

### Cell migration and invasion assays

To assess migration and invasion tendencies, we used transwell chambers to assess migration and invasion, and transwells coated with growth factor-reduced Matrigel (TargetMol, Boston, MA, USA) were specifically used for the invasion assay. The invasion and migration assays were initialized by seeding 8 × 10^4^ and 4 × 10^4^ cells, respectively, into the top chamber in medium devoid of FBS. Cells were incubated at 37°C in a 5% CO_2_ environment for different durations: 24 hours for the invasion assay and 12 hours for the migration assay. After the incubation, the cells that successfully migrated through the membrane were carefully isolated and then fixed with 90% methanol, followed by staining with crystal violet. We used a cotton swab to remove any cells remaining in the upper chamber. Once the aforementioned steps were completed, the migrated cells were subjected to air drying, and the final step of quantification was performed.

### Growth curve analysis

The cells were evenly distributed into a 6-well plate, with each well receiving a seeding density of 2.5 × 10^5^ cells. Following seeding, the cells were treated with 100 μM AG490. The rate of cell proliferation was subsequently determined using a hemocytometer, which enabled us to count the cells at various time intervals, facilitating the construction of a comprehensive growth curve. Simultaneously, a separate experiment in which the cells were systematically distributed into a 96-well plate was performed by ensuring an equal cell suspension volume of 100 μl per well. Each well was seeded with either 2500 or 5000 cells, with a select group of wells exposed to treatment with OTS964 (25 nM). The final stage of the procedure involved the addition of 10 μl of Cell Counting Kit-8 reagent (TargetMol, Boston, MA, USA) to each well. The utilization of this particular kit allowed an assessment of cell viability by recording the absorbance at a wavelength of 450 nm.

### Tube formation assay and ELISA of VEGF levels

Following successful transfection, the 786-O and ACHN cell lines were cultured in freshly prepared complete media for 24 hours. After culture, the supernatants were collected as conditioned media and used in two distinct assays. For the primary assay, a 48-well plate was precoated with a layer of Matrigel. Once the Matrigel solidified, which was achieved by maintaining a constant temperature of 37°C, a suspension of 40,000 hUVECs was prepared using 100 ml of conditioned medium. This mixture was then incubated under the same temperature conditions for a total of 4 hours. After the incubation period, capillary-like tubes were carefully observed using an Olympus DP80 microscope at 200× magnification in five randomly selected fields. The secondary assay was designed to determine the concentration of VEGF present in the conditioned media obtained from the transfected 786-O and ACHN cells using ELISA kits (R&D Systems, MN, USA). The assay was executed in strict adherence to the manufacturer’s defined protocols to ensure the accuracy of the results.

### Transcriptomic and proteomic analyses

RNA was extracted from the RCC cell cultures using a TRIzol reagent kit (Invitrogen, Carlsbad, CA, USA). The integrity of the extracted RNA was validated using an Agilent 2100 bioanalyzer (Agilent Technologies, Santa Clara, CA, USA). Oligo (dT) beads were used to isolate polyA mRNA from the overall RNA. The harvested mRNA was subsequently fragmented into smaller pieces, which were then reverse transcribed into cDNA with random primers. We sequenced the freshly assembled library on the Illumina HiSeq^TM^ 2500 platform (Illumina, San Diego, CA, USA), facilitated by the expertise of Gene Denovo Biotechnology Co. (Guangzhou, China). For the purpose of quantifying transcript expression, we deployed RSEM software. Transcripts that exhibited an absolute fold change greater than 1.5 and an FDR less than 0.05 were labeled as differentially expressed. These genes were then earmarked for a more comprehensive investigation using GO analysis.

In the experimental process, we lysed the RCC cell lines using a designated lysis buffer. The lysates were vortexed to facilitate homogeneous mixing, after which the samples were incubated on ice for half an hour. The subsequent steps involved homogenizing and centrifuging the samples to isolate diverse components based on their densities. After this separation process, we utilized TCEP to reduce disulfide bonds and iodoacetamide to prevent their reformation. The proteins, now in their reduced state, were digested with trypsin, facilitating their breakdown into peptides. These digested proteins were labeled with TMT tags according to the manufacturer’s instructions. After labeling, we separated the peptide mixture at high pH using an Ultimate 3000 UPLC system. An Easy-nLC 1000 system linked to a Q Exactive Hybrid Quadrupole-Orbitrap system further refined our peptide analysis. Proteins were identified with Proteome Discoverer 1.2 along with the Mascot search engine (Matrix Science, London, UK). Proteins showing significant changes, as reflected by fold changes greater than 1.5 and a *p* value less than 0.05, were considered differentially expressed. Using the KEGG database, we annotated these proteins to decipher their functional roles.

### Tail vein injection to assess metastasis in vivo

In our investigation of metastasis, NOD/SCID mice were randomly allocated into two distinct groups of six individuals each. Every mouse in these groups received an intravenous injection of 3 × 10^6^ luciferase lentivirus-infected 786-O cells. These cells were either stable controls or those overexpressing RSK4. The cells were injected into the tail vein in 0.2 ml of PBS. After six weeks, the mice were anesthetized with pentobarbital sodium to prepare them for the next phase of the study. Bioluminescence imaging was performed according to the manufacturer’s protocols (Night OWL II LB983; Berthold Technologies, Bad Wildbad, Germany) to assess the degree and spread of metastasis within these mice. The final phase of the experiment focused on documenting the lifespan of the mice. This assay allowed us to analyze the impact of RSK4 overexpression on survival in the context of metastatic progression.

## Statistical analysis

All the statistical evaluations were performed using SPSS, version 16.0, from SPSS Inc., Chicago, IL, USA. The significance level was set at a two-sided *P* value <0.05.

## Data Availability

The data generated in the present study may be requested from the corresponding author.
